# Supplement of Clinical Practice Guidelines for Endovenous Thermal Ablation for Varicose Veins: Overuse for the Inappropriate Indication

**DOI:** 10.3400/avd.ra.21-00006

**Published:** 2021-12-25

**Authors:** Makoto Mo, Masayuki Hirokawa, Hirono Satokawa, Takumi Yasugi, Takashi Yamaki, Takaaki Ito, Shiro Onozawa, Takashi Kobata, Nozomu Shirasugi, Shintaro Shokoku, Norihide Sugano, Satoru Sugiyama, Katsuyuki Hoshina, on behalf of Guideline Committee, Japanese Society of Phlebology, Tomohiro Ogawa, on behalf of Japanese Commitee of Endovenous Treatment for Varicose Veins

**Affiliations:** 1 Department of Cardiovascular Surgery, Yokohama Minami Kyosai Hospital, Yokohama, Kanagawa, Japan; 2 Ochanomizu Vascular and Vein Clinic, Tokyo, Japan; 3 Department of Cardiovascular Surgery, Fukushima Medical University, School of Medicine, Fukushima, Fukushima, Japan; 4 Department of Cardiovascular Surgery, Ehime University, Toon, Ehime, Japan; 5 Department of Plastic and Reconstructive Surgery, Tokyo Women’s Medical University Medical Center East, Tokyo, Japan; 6 Department of Dermatology, Hyogo College of Medicine, Nisinomiya, Hyogo, Japan; 7 Department of Radiology, Teikyo University Mizonokuchi Hospital, Kawasaki, Kanagawa, Japan; 8 Department of Cardiovascular Surgery, Kanazawa Medical University Himi Municipal Hospital, Himi, Toyama, Japan; 9 Department of Vascular Surgery, Yokohama Asahi Chuo General Hospital, Yokohama, Kanagawa, Japan; 10 Shokoku Shintaro Clinic, Okayama, Okayama, Japan; 11 Department of Surgery, Tokyo Metropolitan Health and Medical Treatment Corporation Ohkubo Hospital, Tokyo, Japan; 12 Department of Surgery, Hiroshima Teishin Hospital, Hiroshima, Hiroshima, Japan; 13 Department of Vascular Surgery, Tokyo University, Tokyo, Japan; 14 Department of Cardiovascular Surgery, Fukushima Dai-Ichi Hospital, Fukushima, Fukushima, Japan

**Keywords:** varicose veins, endovascular surgery, endovenous thermal ablation, guideline, overuse

## Abstract

While endovenous thermal ablation (ETA) become first choice of treatment for varicose veins, overuse of ETA for the inappropriate indication is growing problem. ETA is performed not only on varicose cases without symptom but also non diseased cases with segmental reflux of saphenous veins or no reflux. Indications of ETA was demonstrated in “the Clinical Practice Guidelines for ETA for Varicose Veins 2019” by Japanese Society of Phlebology. Purpose of this supplement is description of basics of correct indication for ETA. We also demonstrate the typical case of overuse of ETA for wrong indication. (This is a translation of Jpn J Phlebol 2020; 31: 39–43.)

## Introduction

Since its approval for national health insurance coverage in 2011, the use of endovenous thermal ablation (ETA) for varicose veins of the lower extremities has become widespread due to its minimal invasiveness, currently accounting for approximately 90% of surgical procedures for lower extremity varicose veins in Japan.^1)^ However, while several patients have benefited from ETA, it is performed for partial valvular insufficiency of the saphenous vein without stasis symptoms and even for normal veins at some medical institutions. In addition, simultaneous treatment of both lower limbs at a higher frequency than that expected epidemiologically (usually 10–20%) and planned repetitive procedures on the same lower limb are performed at these institutions. Amid such situations, the Japanese Committee for Endovascular Treatment for Varicose Veins has alerted implementing surgeons and supervisory doctors to perform appropriate treatment.

In 2019, the Japanese Society of Phlebology published the “2019 Guidelines for Endovenous Thermal Ablation for Lower Extremity Varicose Veins” (ETA Guidelines), consisting of recommendations based on the common clinical questions on ETA selected by the guideline committee and evidence,^2)^ describing appropriate ETA indications. These indications, including the appropriate ranges of the venous diameter and valvular insufficiency, as defined in the previous edition, were considered self-explanatory with the widespread use of ETA and were not specified in the 2019 guidelines. However, the number of improperly treated cases has increased as mentioned above, with inexperienced doctors getting involved in the treatment. Therefore, an addendum describing more basic surgical indications was preferred by many clinicians and insurance certification committee doctors. Under the circumstances described above, the addendum to the guidelines was created to present the basic surgical indications and the details of inappropriate treatment cases, to perform appropriate ETA. Additionally, the indications for cyanoacrylate venous closure, approved in December 2019, are similar to those of ETA, with similar cases not indicated for both modalities.

## Cases Not Indicated for ETA (Cases in Which ETA Should Not Be Performed)

The addendum to the guidelines recommends not performing ETA for the cases listed in **Table 1**. In addition, the national health insurance should not cover ETA for those cases unless there are special circumstances. The reasons are listed below.

**Table 1:** Table 1 Cases where endovenous thermal ablation should not be performed (improper cases)

Normal vein (saphenous vein without valvular insufficiency)
Venous dilatation without valvular insufficiency
Partial valvular insufficiency of the saphenous vein
Primary varicose veins of the lower extremities without stasis symptoms
To prevent worsening of varicose veins or pulmonary thromboembolism in the future

## ETA Should Not Be Performed for Normal Veins or Venous Dilatation without Valvular Insufficiency

According to the guidelines, ETA is indicated for symptomatic primary varicose veins of the lower extremities, and the treatment target veins include the great saphenous vein (GSV), small saphenous vein (SSV), and accessory saphenous vein. According to the mechanism of development, varicose veins of the lower extremities are classified into primary, secondary, and congenital. Primary varicose veins of the lower extremities are defined as those developing due to valvular insufficiency of the superficial venous system, mostly the saphenous vein.^3)^ The presence or absence of valvular insufficiency should always be evaluated using ultrasonography, and is identified if the reflux time measured by pulsed Doppler through blood flow induction in a standing or sitting position exceeds 0.5 s.^4)^ The previous edition of the ETA guidelines recommended measuring venous diameters and indicating ETA if the mean diameter of the saphenous vein was 4 mm or greater. However, the recommended saphenous vein diameter was not stated in the 2019 guidelines as it varies between GSV and SSV and depends on the physique; i.e., it can only be an indicator for the blood reflux volume. If the diameter is 4 mm or smaller, symptoms are often mild even with reflux. Therefore, to determine the indication for ETA, thorough consideration and detailed examination is necessary. Also, if there is no valvular insufficiency in the saphenous vein, regardless of the diameter, it is not primary varicose vein. Therefore, even if venous dilatation with a diameter of 4 mm or greater exists, or spider or reticular veins and edema classified into C1 and C3 of the Clinical Etiological Anatomical Pathophysiological Classification (CEAP)^5)^ exist, there is no indication for ETA if valvular insufficiency in the saphenous vein is absent.

Performing ETA on the saphenous vein without valvular insufficiency would cause unnecessary invasion, leading to the loss of an alternative graft option for future cardiac or lower limb bypass surgery. Therefore, “patients should not be harmed,” as such an act is against the “medical ethics” and must be strictly avoided.

## ETA Should Not Be Performed for Partial Saphenous Vein Valvular Insufficiency (**Fig. 1**)

**Figure 1:**
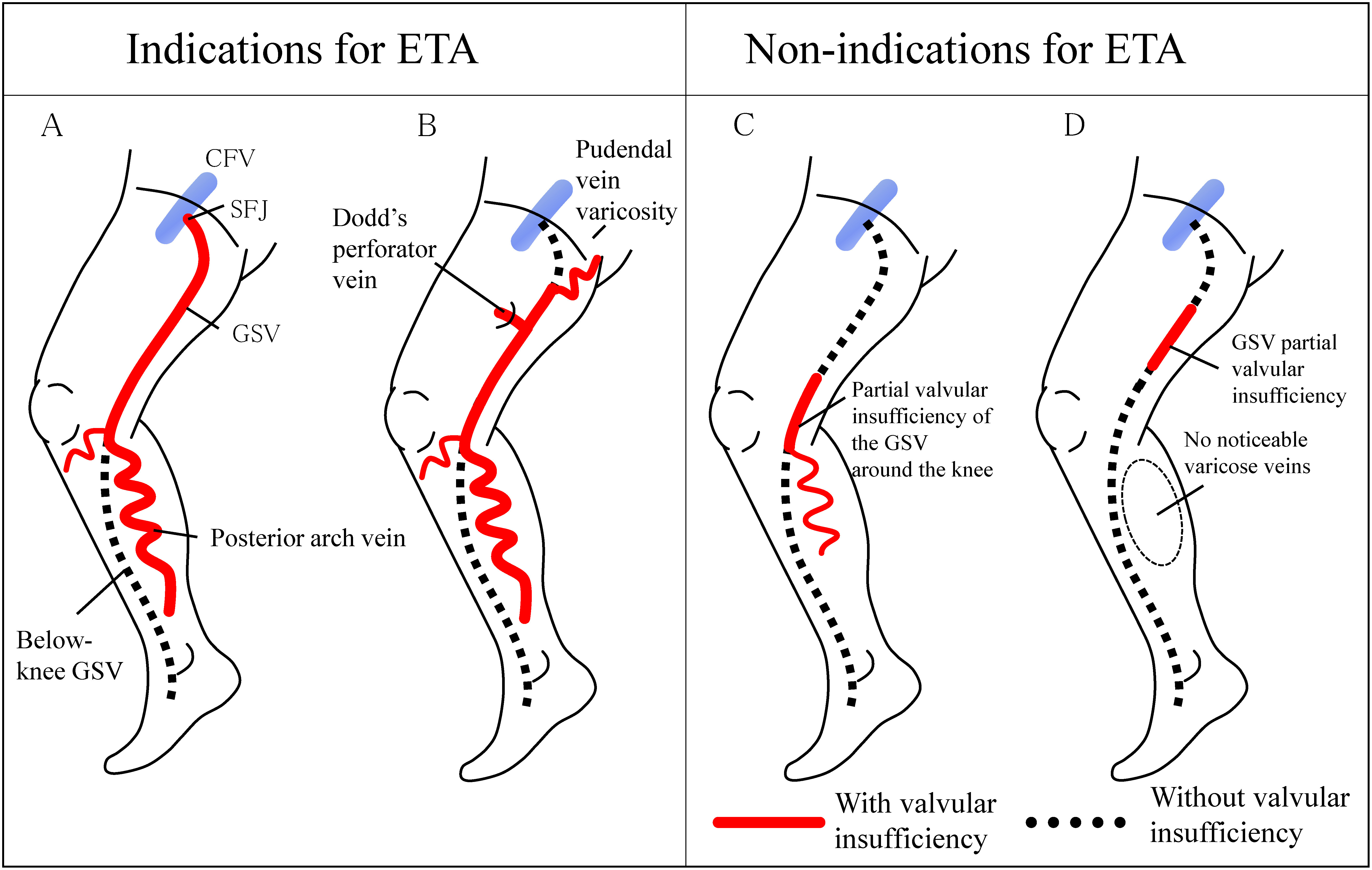
Fig. 1 Valvular insufficiency of the saphenous vein and indications for endovenous thermal ablation (ETA).

Valvular insufficiency of the saphenous vein spanning a wide area from the SFJ to below the knee (**A**) and pudendal vein varicosity without SFJ insufficiency and valvular insufficiency of the GSV with Dodd’s perforator vein serving as a reflux source (**B**) are ETA indications. However, neither localized valvular insufficiency of the GSV around the knee (**C**) nor partial GSV insufficiency without tributary varicose veins (**D**) is an indication for ETA. SFJ: sapheno-femoral junction; CFV: common femoral vein; GSV: great saphenous vein

Although ultrasonography reveals valvular insufficiency in superficial veins in 35%–43% of healthy individuals without varicose veins, most of these are partial valvular insufficiency.^6,7)^ Partial valvular insufficiency does not cause symptoms, and although blood reflux worsens over time, all patients do not develop chronic venous insufficiency.^6–8)^ To date, clinical studies of ETA for lower extremity varicose veins have been conducted in patients with symptomatic lower extremity varicose veins,^9,10)^ including mostly axial reflux cases associated with extensive saphenous vein valvular insufficiency ranging from the sapheno-femoral junction (SFJ) to below the knee (**Fig. 1A**). No study has included a segmental reflux case due to localized valvular insufficiency of the GSV around the knee, common in mild saphenous venous reflux (**Figs. 1C** and **1D**). Most mild varicose vein cases due to localized valvular insufficiency are asymptomatic without treatment requirement.^11,12)^ Even symptomatic cases are not indicated for ETA but for varicose vein resection or sclerotherapy. In rare cases, the medial perforating branch of the femoral vein (Dodd’s perforator vein) or pudendal varicose vein serves as a reflux source and causes valvular insufficiency of the GSV without SFJ insufficiency. These cases are indications for ETA (**Fig. 1B**). In atypical cases, a detailed ultrasonographic examination should be performed before surgery to thoroughly examine the characteristics of the reflux source and the extent to which the reflux source is contributing to the valvular insufficiency of the GSV, explaining the results to the patient.

## ETA Should Not Be Performed for Primary Varicose Veins of the Lower Extremities without Symptoms of Stasis

Symptoms of varicose veins of the lower extremities include feeling of heaviness, dullness, muscle cramping, itching, edema, pain, and discomfort of the lower extremities^13)^ as well as skin symptoms such as eczema, lipodermatosclerosis, and venous stasis ulcer. ETA is performed in patients with varicose veins of the lower extremities to “alleviate the symptoms.” Patients with valvular insufficiency of the saphenous vein spanning a wide area from the SFJ to below the knee are not indicated for ETA if there are no stasis symptoms. Symptoms of varicose veins of the lower extremities are not disease-specific and are often caused by other diseases. Thus, the patient’s presenting symptoms should be rigorously evaluated for lower extremity varicose veins and other possible causes should be ruled out, before determining the surgical indication. To evaluate whether the symptoms are due to venous stasis, the information, such as whether the symptoms worsen in the standing position or in the evening rather than in the morning, and whether wearing elastic stockings alleviate the symptoms, should be used as reference. In patients with spider/reticular varicose veins without visibly apparent tributary varicose veins, valvular insufficiency in the saphenous vein is unlikely to be associated with the symptoms.^8)^

## ETA Should Not Be Performed to Prevent Worsening of Varicose Veins or Pulmonary Thromboembolism in the Future

Observation of the natural course of patients with varicose veins of the lower extremities shows progression of venous reflux and symptoms over time; however, only limited cases require surgical treatment.^14)^ ETA is not indicated for mild varicose veins for preventing progression, as it is a benign disease, and studies to date have shown a favorable prognosis with treatment after the appearance of symptoms.^9,10)^

Varicose veins of the lower extremities sometimes coincide with deep vein thrombosis (DVT) and pulmonary thromboembolism (PTE). However, while the prevalence of varicose veins of the lower extremities is high, the incidence of PTE is low. A recent report analyzing health insurance data showed that the incidence of DVT is 5.3 times and that of PTE 1.7 times higher in patients who were diagnosed with varicose veins of the lower extremities.^15)^ However, the analysis was based on the disease names for health insurance lacking the accuracy of diagnosis, and patients with varicose veins of the lower extremities undergo lower extremity venous ultrasonography examinations at a high frequency, influencing the analysis results. On the contrary, DVT/PTE can rarely occur as a complication after ETA.^16)^ Therefore, there is no evidence of the benefits of invasive procedures for lower extremity varicose veins, including sclerotherapy and stripping surgery, for the prevention of DVT/PTE and ETA should not be performed.

## A Case of Inappropriate Treatment

A typical case of inappropriate treatment is shown below. This case has been reconstructed as a representative case based on the one that was provided and reported to the Japanese Society of Phlebology. It has been modified for protection of personal information and does not refer to a specific individual patient or medical institution.

**Figure 2:**
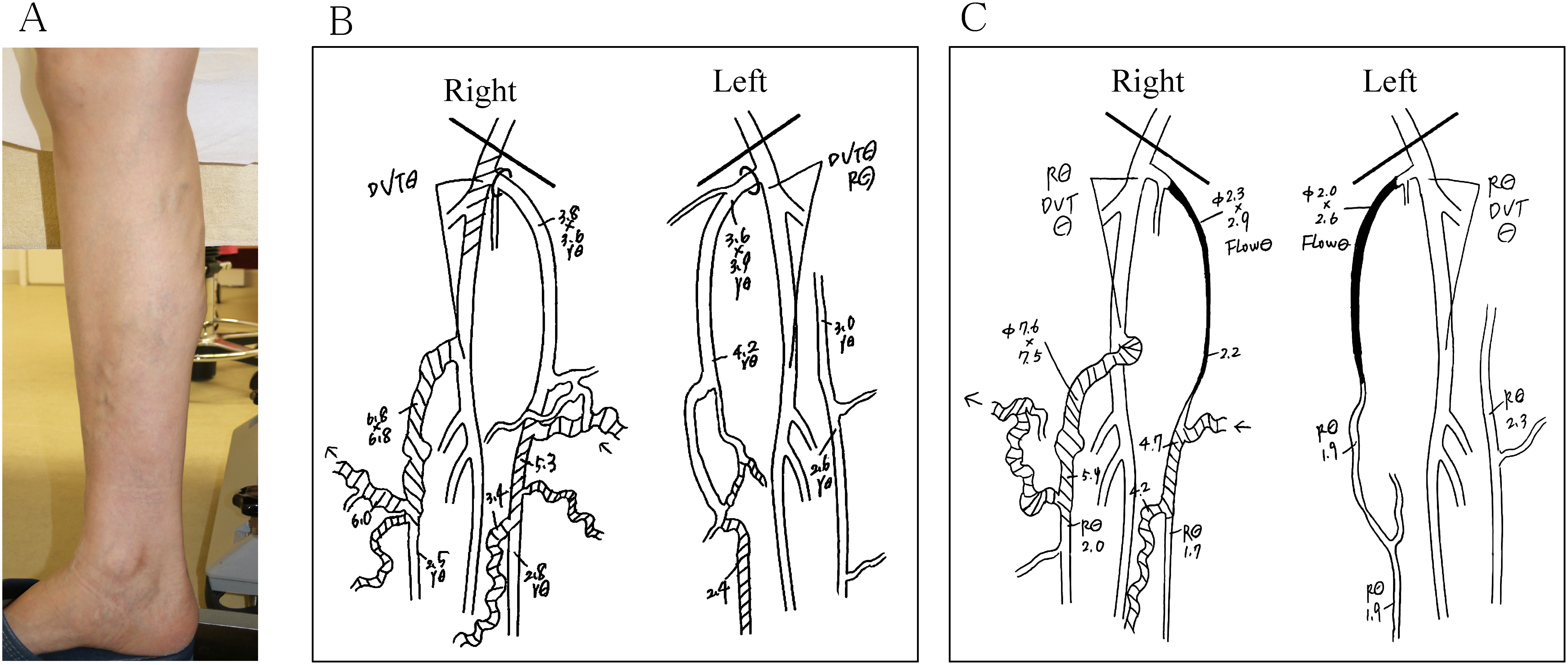
Fig. 2 A case of improper treatment.

The patient was a woman in her 50s who visited a medical institution with a chief complaint of varicose veins, fatigability, and edema of the right lower limb. Ultrasonography of the lower extremity veins revealed valvular insufficiency in the right SSV and femoral vein with left distal GSV, and right SSV dilatation with a diameter of 6.8×6.8 mm, diagnosed as an indication for ETA on the right SSV (**Fig. 2**). After two months, the patient visited the second institution and was also recommended treatment of the right limb. She was also offered ETA of her left lower limb for a preventive purpose. ETA on bilateral GSV was performed at the second institution, which did not improve the symptoms. Therefore, seven months after surgery, the patient went back to the first institution she originally visited. The lower extremity venous ultrasonography examination revealed ETA performed on the bilateral GSV, but no evidence of ETA on the right SSV. There were primarily two problems with this case. First, although valvular insufficiency on the right SSV was evidenced by lower extremity venous ultrasonography, it was left untreated (they were probably unable to diagnose it). Secondly, bilateral lower extremity ETA seemed to be performed routinely regardless of the presence or absence of valvular insufficiency.

(**A**) Macroscopic findings: Lower extremity varicose veins are seen on the medial side of the right lower limb. (**B**) Venous ultrasonography findings at the first examination: Valvular insufficiency is seen in the right SSV and femoral vein and left distal GSV. Dilatation is seen in the right SSV with a diameter of 6.8×6.8 mm. The shaded area indicates the range of valvular insufficiency. (**C**) Venous ultrasonography findings at the reexamination: Bilateral GSV is occluded by ETA. Valvular insufficiency and dilatation of the right SSV expanded to a diameter of 7.6×7.5 mm. SSV: small saphenous vein; GSV: great saphenous vein; DVT: deep vein thrombosis

## Conclusion

Although we were aware of a small number of doctors providing improper treatment, we believed that the approval of the health insurance coverage of ETA would eliminate inappropriate treatment in ETA. However, recently improper treatment has been provided more systemically, on a larger scale. Even though the majority of doctors adhere to the guidelines and sincerely provide proper medical care to their patients, a small number provide improper treatment due to ignorance or financial motivation, affecting the budget of healthy health care services under public health insurance. We prepared this ETA guideline addendum to improve the situation. This article concludes with the Hippocratic Oath related to the ethics of medicine.

“I will apply dietetic and lifestyle measures for the benefit of the sick to the best of my ability and judgment and will keep them from harm and injustice.”^17)^

## Notes

This is a translation of Jpn J Phlebol 2020; 31: 39–43.

## References

[1] 4th NBD Open Data, Ministry of Health, Labour and Welfare. [https://www.mhlw.go.jp/stf/seisakunitsuite/bunya/0000177221_00003.html] (Accessed on January 3, 2020)

[2] Hirokawa M, Satogawa H, Yasugi T, et al. Guidelines for endovenous thermal ablation for lower extremity varicose veins. Phlebology 2019; 30 **Suppl**: i-81.

[3] Obitsu Y. Pathology of varicose veins of the lower extremities. In: Japanese Society of Phlebology ed. New Textbook of Clinical Phlebology. Tokyo: Medical View, 2019: 198-202. (in Japanese)

[4] Labropoulos N, Tiongson J, Pryor L, et al. Definition of venous reflux in lower-extremity veins. J Vasc Surg 2003; 38: 793-8. 14560232 10.1016/s0741-5214(03)00424-5

[5] Eklöf B, Rutherford RB, Bergan JJ, et al. Revision of the CEAP classification for chronic venous disorders: consensus statement. J Vasc Surg 2004; 40: 1248-52. 15622385 10.1016/j.jvs.2004.09.027

[6] Maurins U, Hoffmann BH, Lösch C, et al. Distribution and prevalence of reflux in the superficial and deep venous system in the general population—results from the Bonn Vein Study, Germany. J Vasc Surg 2008; 48: 680-7. 18586443 10.1016/j.jvs.2008.04.029

[7] Robertson LA, Evans CJ, Lee AJ, et al. Incidence and risk factors for venous reflux in the general population: Edinburgh Vein Study. Eur J Vasc Endovasc Surg 2014; 48: 208-14. 24951373 10.1016/j.ejvs.2014.05.017

[8] Wrona M, Jöckel KH, Pannier F, et al. Association of venous disorders with leg symptoms: results from the Bonn Vein Study 1. Eur J Vasc Endovasc Surg 2015; 50: 360-7. 26141786 10.1016/j.ejvs.2015.05.013

[9] Lurie F, Creton D, Eklof B, et al. Prospective randomized study of endovenous radiofrequency obliteration (closure procedure) versus ligation and stripping in a selected patient population (EVOLVeS Study). J Vasc Surg 2003; 38: 207-14. 12891099 10.1016/s0741-5214(03)00228-3

[10] Rasmussen LH, Bjoern L, Lawaetz M, et al. Randomized trial comparing endovenous laser ablation of the great saphenous vein with high ligation and stripping in patients with varicose veins: short-term results. J Vasc Surg 2007; 46: 308-15. 17600655 10.1016/j.jvs.2007.03.053

[11] Eklof B, Perrin M, Delis KT, et al. Updated terminology of chronic venous disorders: the VEIN-TERM transatlantic interdisciplinary consensus document. J Vasc Surg 2009; 49: 498-501. 19216970 10.1016/j.jvs.2008.09.014

[12] Labropoulos N, Leon M, Nicolaides AN, et al. Superficial venous insufficiency: correlation of anatomic extent of reflux with clinical symptoms and signs. J Vasc Surg 1994; 20: 953-8. 7990191 10.1016/0741-5214(94)90233-x

[13] Khilnani NM, Grassi CJ, Kundu S, et al. Multi-society consensus quality improvement guidelines for the treatment of lower extremity superficial venous insufficiency with endovenous thermal ablation from the Society of Interventional Radiology, Cardiovascular Interventional Radiological Society of Europe, American College of Phlebology and Canadian Interventional Radiology Association. J Vasc Interv Radiol 2010; 21: 14-31. 20123189 10.1016/j.jvir.2009.01.034

[14] Kostas TI, Ioannou CV, Drygiannakis I, et al. Chronic venous disease progression and modification of predisposing factors. J Vasc Surg 2010; 51: 900-7. 20347686 10.1016/j.jvs.2009.10.119

[15] Chang SL, Huang YL, Lee MC, et al. Association of varicose veins with incident venous thromboembolism and peripheral artery disease. JAMA 2018; 319: 807-17. 29486040 10.1001/jama.2018.0246 PMC5838574

[16] Nemoto H, Mo M, Ito T, et al. Venous thromboembolism complications after endovenous laser ablation for varicose veins and role of duplex ultrasound scan. J Vasc Surg Venous Lymphat Disord 2019; 7: 817-23. 31540837 10.1016/j.jvsv.2019.06.014

[17] Emoto H. Hippocrates and medical ethics, Japan Medical Association. [http://dl.med.or.jp/dl-med/doctor/member/kiso/k3.pdf] (Accessed on January 5, 2020)

[18] Mo M, Hirokawa M, Satokawa H, et al. Supplement of clinical practice guidelines for endovenous thermal ablation for varicose veins: overuse for the inappropriate indication. Jpn J Phlebol 2020; 31: 39-43. (in Japanese) 10.3400/avd.ra.21-00006 PMC8752913 35082936

